# Infrared Spectroscopy of Li^+^ Solvation in Diglyme: Ab Initio Molecular Dynamics and Experiment

**DOI:** 10.1021/acs.jpcb.3c05612

**Published:** 2023-10-11

**Authors:** Fangyong Yan, Kallol Mukherjee, Mark Maroncelli, Hyung J. Kim

**Affiliations:** † Department of Chemistry, Carnegie Mellon University, Pittsburgh, Pennsylvania 15213, United States; ‡ Department of Chemistry, The Pennsylvania State University, University Park, Pennsylvania 16802, United States

## Abstract

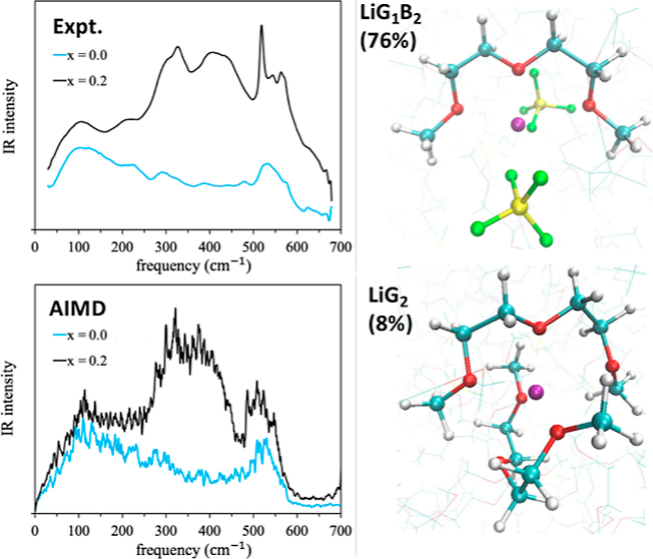

Infrared (IR) spectra of solutions of the lithium salt LiBF_4_ in diglyme, CH_3_O(CH_2_CH_2_O)_2_CH_3_, are studied via IR spectroscopy and ab initio molecular dynamics (AIMD) simulations. Experiments show that the major effects of LiBF_4_, compared to neat diglyme, are the appearance of a new broad band in the 250–500 cm^–1^ frequency region and a broadening and intensity enhancement of the diglyme band in the 900–1150 cm^–1^ region accompanied by a red-shift. Computational analysis indicates that hindered translational motions of Li^+^ in its solvation cage are mainly responsible for the new far-IR band, while the changes in the mid-IR are due to Li^+^-coordination-dependent B–F stretching vibrations of BF_4_^–^ anions coupled with diglyme vibrations. Molecular motions in these and lower frequency regions are generally correlated, revealing the collective nature of the vibrational dynamics, which involve multiple ions/molecules. Herein, a detailed analysis of these features via AIMD simulations of the spectrum and its components, combined with analysis of the generalized normal modes of the solution components, is presented. Other minor spectral changes as well as diglyme conformational changes induced by the lithium salt are also discussed.

## 1 Introduction

Glymes are a family of liquid ethers, CH_3_O(CH_2_CH_2_O)_*n*_CH_3_ (*n* = 1, 2, ··), which are chemically inert, biodegradable, and mostly miscible with water and hydrocarbon solvents. Their ether oxygen atoms effectively coordinate cations, resulting in the efficient dissociation of ion pairs in these solvents. As such, they are widely used as reaction media for a wide variety of different chemical processes. They also provide a safer and environmentally more friendly alternative to organic solvents, such as carbonates, for use as an electrolyte solution in lithium-ion batteries.^1,2^ Furthermore, some of their mixtures with salts, especially lithium salts at relatively high concentrations, show properties similar to those of ionic liquids (ILs), including low melting points below 100 °C and negligible vapor pressures, offering a new class of ionic materials (“solvate ILs”).^3−5^

Due to their importance in energy storage, solutions of various lithium salts in low-molecular-weight glymes and in poly(ethylene oxide)s (PEOs) in both the liquid and crystal phases have been a subject of intensive experimental investigation for over three decades.^1,2^ Various spectroscopic methods have been employed to characterize their structure and dynamics, especially the influence of added lithium salts.^6−16^ It was found that glymes and PEOs complex strongly to Li^+^ ions via their ether oxygen atoms, thereby forming a variety of Li^+^-solvates.^6−9,12,13,15,16^ One important manifestation of this complexation is the appearance of a broad infrared (IR) band in the 250–500 cm^–1^ frequency region,^6,9−12^ attributed to ion-cage vibrations,^6^ i.e., rattling motions of Li^+^ in its solvation cage. This far-IR feature is shared by Li^+^ solutions in many other organic solvents^17−19^ and in ILs.^20,21^ Another signature of Li^+^–glyme and Li^+^–PEO complexation is the development of a new Raman band between ∼860 and 880 cm^–1^.^6,7,9,12,16^ This band is attributed to symmetric Li^+^–ether oxygen vibrations, referred to as breathing modes, originally proposed for metal–crown ether complexes.^22^

In parallel, a great deal of computational effort, primarily via molecular dynamics (MD) simulations, has been directed toward a detailed understanding of Li^+^–PEO and Li^+^–glyme mixtures.^23−38^ Li^+^ solvation structure as well as transport and related dynamics, which require prolonged simulations, were studied mainly using classical MD.^24,25,27−35,38^ Ab initio MD (AIMD) was also employed in recent study of Li^+^ solvation structure in glymes^36,37^ and in the analysis of Li^+^ transport along a single PEO chain.^26^ Various properties of Li^+^ solvates, such as coordination structure, glyme backbone conformations, and normal mode vibrations at local energy minima, have also been analyzed with ab initio methods.^12,36−45^ However, the vibrational and spectroscopic properties of Li^+^–PEO and Li^+^–glyme mixtures have not been simulated previously via MD.

In this article, we investigate far- and mid-IR properties of solutions of LiBF_4_ in diglyme, CH_3_O(CH_2_CH_2_O)_2_CH_3_, as well as those of the neat diglyme. Changes induced by the addition of LiBF_4_ are examined in detail by using AIMD and vibrational spectroscopy. To the best of our knowledge, this is the first AIMD study of the IR properties of pure glymes and their mixtures with lithium salts. Combining different analyses, viz., decomposing the spectral features into auto- and cross-correlations of component currents^21,46^ and calculating solution-phase generalized normal modes (GNMs),^47,48^ we obtain molecular details of the mixture spectrum, including mechanisms for the spectral changes caused by the Li salt. It is found that Li^+^ translational dynamics coupled with vibrations of the surrounding cage are responsible for the broad far-IR band between 250 and 500 cm^–1^. The primary mid-IR band, found in the 900–1150 cm^–1^ region, exhibits a broadening and intensity enhancement on its red side, arising mainly from the B–F stretching vibrations of BF_4_^–^, coupled with diglyme vibrations. Structural analysis shows that backbone conformations of diglyme molecules are significantly altered by complexation to Li^+^ in the mixture compared to neat diglyme. Details of these findings are presented in Section 3, while the experimental and computational methods employed in our study are explained in Section 2. Section 4 concludes.

The present work extends our previous work on LiBF_4_ solutions in propylene carbonate (PC) and the IL 1-ethyl-3methylimidazolium tetrafluoroborate (EmimBF_4_).^21^ Some of the key spectral changes observed in the present work are shared by the LiBF_4_ + PC system, although the details are different. Hereafter, this system will be referred to as the PC mixture and the LiBF_4_ + EmimBF_4_ system as the IL mixture.

## 2 Methods

### 2.1 Theory and Computational Methods

IR properties of two systems were studied via AIMD: pure diglyme and its 4:1 mixture with LiBF_4_ (Table 1). The simulation protocols and IR calculation methods employed are the same as in our prior study of the IL and PC solutions of LiBF_4_.^21^ Therefore, only a very brief account of the computational methods is given here. For details, the reader is referred to ref (21). The BLYP functional^49,50^ with the DFT-D3 dispersion correction^51^ was employed using the all-electron Gaussian augmented plane wave method^52^ with the 6-311G(d,p) basis set^53^ and a plane wave cutoff of 280 Ry. AIMD simulations were performed in the canonical ensemble at 350 K with a time step of 0.5 fs using the Quickstep module of CP2K.^54^ Each system was placed in a cubic box with periodic boundary conditions applied and the Nosé–Hoover thermostat^55−57^ employed to control its temperature. Because of the disordered nature of our systems, only the Γ-point of the Brillouin zone was considered in the electronic description of the simulations.^58,59^ Three trajectories were simulated, each for at least 70 ps for neat diglyme and 120 ps for the mixture, preceded by equilibration for 10 ps of equilibration.

**Table 1:** AIMD Simulations

system^a^	no. of trajectories	density (g/cm^3^)	equilibration time (ps)	total production time (ps)^b^
16 diglyme	3	0.884	10	231
16 diglyme + 4 LiBF_4_	3	0.977	10	362

a Numbers indicate the number of either diglyme molecules or ion pairs.

b Sum of the production times of three independent trajectories.

The initial configurations for AIMD were obtained from classical MD simulations using the Gromacs program.^60^ The system densities were determined in the isobaric isothermal ensemble at 1 bar and 350 K. For these density determinations, neat diglyme, consisting of 500 diglyme molecules, and the mixture consisting of 500 diglyme molecules + 125 LiBF_4_ ion pairs were simulated. Optimized potentials for liquid simulations (OPLS) parameters^61,62^ were employed for Li^+^ and diglyme while the parameters for BF_4_^–^ were taken from ref (63). A Nosé–Hoover thermostat^55−57^ and a Parrinello–Rahman barostat^64,65^ were used to control the system temperature and pressure. Using the densities thus determined, smaller classical systems, identical in the composition to the corresponding AIMD systems, were constructed and simulated in the canonical ensemble at 350 K. The final configurations of these classical MD runs were then geometry optimized with Quickstep and used as the initial configurations for AIMD.

IR spectra were calculated from the Fourier transform of the time correlation function of the system dipole moment .^66^ With desymmetrization of the classical time correlation function to meet the detailed balance condition,^67,68^ the volume-normalized IR absorption cross section α(ω) at angular frequency ω is given by^21,58,66^
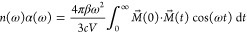
1
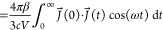
2
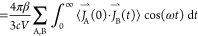
3where *n*(ω) is the index of refraction of the medium, *V* is the volume of the system, β is the inverse temperature measured in units of Boltzmann’s constant, *c* is the speed of light in vacuum, (*t*) [= (d(*t*)/d*t*)] is the current density integrated over *V*, A and B denote ionic/molecular species (A, B = Li^+^, BF_4_^–^, and diglyme), and ⟨···⟩ represents an ensemble average. Since  is well-defined for individual ions while  is not, eq 3 provides a convenient framework for analyzing IR contributions from different ionic species.^46^ The electronic contribution to  for individual component species was calculated by localization of electronic charges using maximally localized Wannier functions (MLWFs).^69−71^

We also studied quenched normal modes (QNMs) and GNMs. In QNM analyses, four configurations were selected from each of the three AIMD trajectories, geometry optimized, and normal mode calculations performed on these optimized structures using CP2K. The absorption coefficient *A*_*j*_ for normal mode *j* was calculated via^72,73^
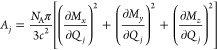
4where *N*_A_ is Avogadro’s number, *Q*_*j*_ is the *j*th normal mode, and *M*_*x*,*y*,*z*_ are the *x*, *y*, and *z* components of . A continuous IR spectrum was obtained from the *A*_*j*_’s by introducing a Lorentzian line shape function with a line width of 5 cm^–1^ for each normal mode and summing over all modes.

GNMs were calculated from the power spectra of the velocity time correlations of the constituent atoms of component species, following the approach described in refs (47), (48) and (74) using the TRAVIS program.^75,76^ GNMs represent the average intramolecular vibrations of single ions or molecules in the liquid phase around their reference structures, which are defined by the minima on the potential energy surface of the isolated ion/molecule. It should be noted that the effects of intermolecular interactions and nonlinear vibrational coupling are captured in the GNM calculations but collective vibrations involving two or more ions or molecules are not.

### 2.2 Experimental Methods

The anhydrous salts lithium tetrafluoroborate (98.0%, Acros-Organics) and lithium trifluoromethylsulfonate (“LiTfO”, >98%, TCI America) were used as received. Anhydrous diglyme (2-methoxyethyl ether, 99.5%, Sigma-Aldrich) was dried over molecular sieves. Karl Fisher titration showed the dried diglyme and diglyme + salt mixtures to have water contents between 200 and 500 ppm by mass. Mixtures were prepared by weight in glass vials and stirred until a clear solution was obtained.

Far-infrared spectra (30–680 cm^–1^) were collected in the transmission mode at 4 cm^–1^ resolution by using a Bruker Vertex 80v spectrometer operating with a globar source and dTGS detector. Samples were contained in a temperature-controllable cell (Harrick Scientific, TFC-M13-3) consisting of a pair of polyethylene windows (1 mm thickness and 13 mm diameter) and a Teflon spacer of 6 μm thickness. Mid-infrared spectra were recorded in the attenuated total reflection (ATR) mode at 2 cm^–1^ resolution over the range 500–4000 cm^–1^ by using a Bruker Vertex 70v spectrometer equipped with a diamond ATR cell, a standard MIR source, and a cooled MCT detector. Both instruments were operated under continuous N_2_ purging at room temperature (21 ± 1) °C.

## 3 Results

**Figure 1:**
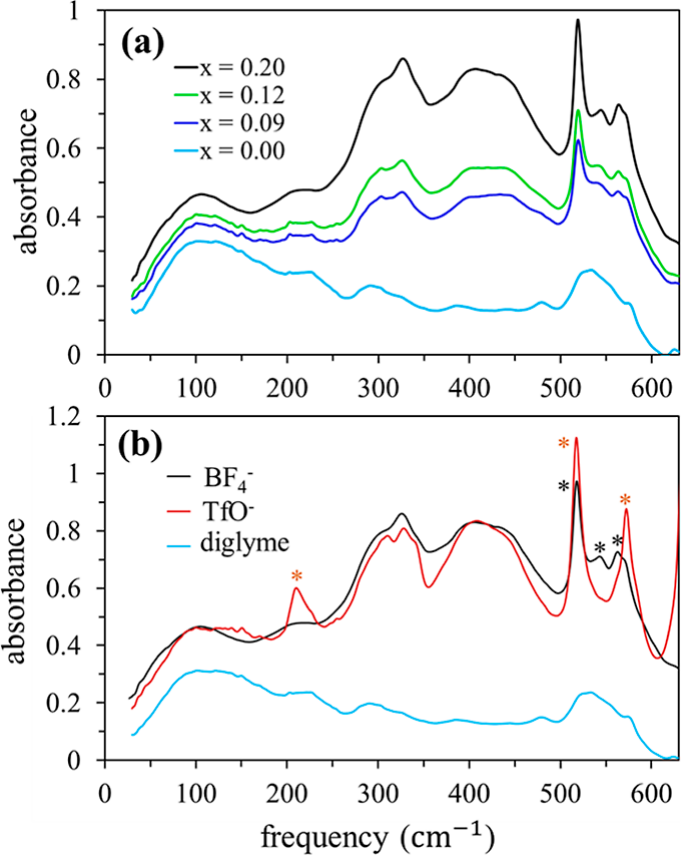
(a) Experimental far-IR spectra of pure diglyme and diglyme + LiBF_4_ mixtures at various LiBF_4_ mole fractions (*x*). (b) Comparison of the experimental spectra of mixtures of diglyme with LiBF_4_ and LiTfO at *x*_Li_ = 0.2. The LiTfO spectra are normalized to the LiBF_4_ spectra at 400 cm^–1^ to aid comparison. Asterisks indicate anion peaks.

As motivation for the computational analysis presented below, experimental results for the far-IR spectra of the diglyme + LiBF_4_ mixtures at different LiBF_4_ concentrations are shown in Figure 1a. The addition of LiBF_4_ to diglyme has a pronounced effect on the IR absorption in the frequency range of 150–650 cm^–1^. As the LiBF_4_ concentration increases, a new broad band with a double-peak structure develops between 250 and 500 cm^–1^ while a distinct multipeak structure appears superimposed on the diglyme band centered around 530 cm^–1^. Figure 1b shows that very similar spectral changes are obtained with another lithium salt, LiTfO (TfO^–^ = CF_3_SO_3_^–^). Although we focus exclusively on diglyme mixtures with LiBF_4_, the LiTfO example in Figure 1 is used to highlight the fact that development of a broad band in the ∼250–500 cm^–1^ region is observed upon addition of various lithium salts to a wide range of different solvents, including organic solvents and ILs, though details of the band structure may differ (see Figure S1 in the Supporting Information).^6,9−12,17−21^ This feature arises primarily from hindered translational motions (“rattling”) of Li^+^ ions in differing solvation environments.^6,9−12,17−21^ In the following section, we thus start with the analysis of Li^+^ solvation, and how it is coordinated by ions and diglyme molecules.

### 3.1 Solvation Structure and Coordination of Li^+^

**Figure 2:**
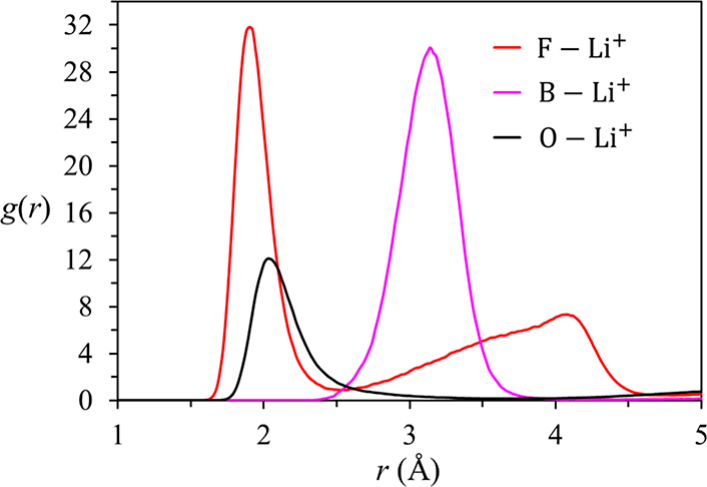
RDFs of F–Li^+^, B–Li^+^, and O–Li^+^ in the diglyme + LiBF_4_ mixture (*x*_LiBF_4__ = 0.2).

Radial distribution functions (RDFs) of F and B atoms of BF_4_^–^ and O of diglyme around Li^+^ are displayed in Figure 2. The pronounced primary peaks of the F–Li^+^ and B–Li^+^ distributions, respectively at 1.92 and 3.14 Å, show strong coordination of Li^+^ by F of BF_4_^–^ anions. The secondary peak in the F–Li^+^ RDF is primarily due to F atoms of coordinating BF_4_^–^ anions that face away from Li^+^. Li^+^ is also strongly coordinated by diglyme, as indicated by the peak at 2.04 Å in the O–Li^+^ distribution. In contrast to the clear minimum at 2.56 Å in the F–Li^+^ RDF, the O–Li^+^ distribution exhibits a broad tail, which reaches only a shallow minimum near 3.6 Å. Also, the main peak of the O–Li^+^ RDF is broader than that of the F–Li^+^ distribution. These differences are due to the conformational flexibility of diglyme, as discussed in Section 3.2 below. The respective peak positions of the F–Li^+^ and O–Li^+^ distributions, 1.92 and 2.04 Å, compare very well with the X-ray diffraction results for the (diglyme)_*n*_/LiBF_4_ (*n* = 1 and 2) crystal structures, 1.9 and 2.05–2.15 Å.^13^

We further characterize Li^+^ solvation in two different ways (Tables 2 and 3). In Table 2, we show probabilities of different numbers of F and O atoms coordinating Li^+^. In the absence of a sharp minimum in the O–Li^+^ RDF, we employ the position of the F–Li^+^ minimum, 2.56 Å, as the cutoff distance to define coordination by both F and O atoms. The results in Table 2 indicate that a variety of Li^+^ coordination shells are present. Nonetheless, more than 70% of the Li^+^ ions are accounted for by 2F + 2O or 2F + 3O coordination (see below). The average number of atoms in the Li^+^-coordination shell is 4.6, composed of 1.7 F and 2.9 O atoms.

**Table 2:** Distribution of Atoms Coordinated to Li^+^

	2F + O	2F + 2O	3F + 2O	1F + 3O	2F + 3O	3F + 3O	4O	1F + 4O	5O	6O
prob. (2.56 Å)	0.6%	33.9%	3.2%	5.8%	38.1%	1.4%	2.1%	7.8%	4.8%	1.4%
prob. (2.70 Å)	0.1%	25.3%	4.7%	3.8%	44.2%	3.5%	1.0%	8.9%	4.7%	2.7%

**Table 3:** Distribution of Molecules and Ions Coordinated to Li^+^

	LiB_2_	LiG_1_	LiG_1_B_1_	LiG_1_B_2_	LiG_2_	LiG_2_B_1_
prob. (2.56 Å)	0.9%	0.3%	14.2%	76.0%	8.1%	0.3%
prob. (2.70 Å)	0.3%	<0.1%	13.4%	77.3%	8.3%	0.6%

In Table 3, Li^+^ solvates are categorized in terms of the number of BF_4_^–^ anions (“*B*”) and diglyme molecules (“*G*”) coordinating each Li^+^ ion. In order to exclude transitory structures, we consider a diglyme molecule to be part of a Li^+^ solvate only when two or more of its O atoms coordinate to the same Li^+^ ion. We label such molecules as bound diglyme (bG). Those with one or no Li^+^-coordinating O atoms are considered unbound diglyme (uG), as done in ref (33). BF_4_^–^, on the other hand, is defined as a coordinating anion if one or more of its F atoms coordinate to Li^+^, as in previous studies.^21,77^ The three most populous Li^+^ complexes are a Li^+^ ion coordinated by one diglyme molecule and two BF_4_^–^ anions (LiG_1_B_2_, 76%) and a Li^+^ ion coordinated either by one diglyme and one BF_4_^–^ anion (LiG_1_B_1_, 14%) or by two diglyme molecules (LiG_2_, 8%). This means that 90% of the Li^+^ solvation cages consist of one diglyme molecule and one or two BF_4_^–^ anions. The presence of the 3F + 2O (3.2%) and 3F + 3O (1.4%) compositions (Table 2), together with a maximum number of two anions in the coordination shell, reveals that about 5% of BF_4_^–^ anions are bidentate ligands, i.e., they form a bifurcated coordination with the central Li^+^ ion.^21,77^ It is interesting to note that the dominant motif of Li^+^ solvation in this solution closely resembles that found in crystalline diglyme–LiBF_4_, where each Li^+^ is coordinated by three O atoms from a diglyme molecule and two F atoms, one each from two BF_4_^–^ anions.^13^

To check how sensitive the above results are to the choice of cutoff, we also considered a 2.70 Å cutoff. Comparison of the results of these two cutoff distances in Tables 2 and 3 demonstrates that while the populations differ in detail, the main characteristics of Li^+^ coordination, such as the most populous coordination complexes and their compositions, remain essentially unaffected. As such, we will use the cutoff distance of 2.56 Å in the definition of Li^+^-coordinating atoms in the remainder of this article. With this cutoff, the ratio of bG to uG populations in the mixture is 1:2.2 (*x*_LiBF_4__ = 0.2).

### 3.2 Dihedral Angle Distribution of Diglyme Molecules in Pure Diglyme and Diglyme + LiBF_4_

**Figure 3:**
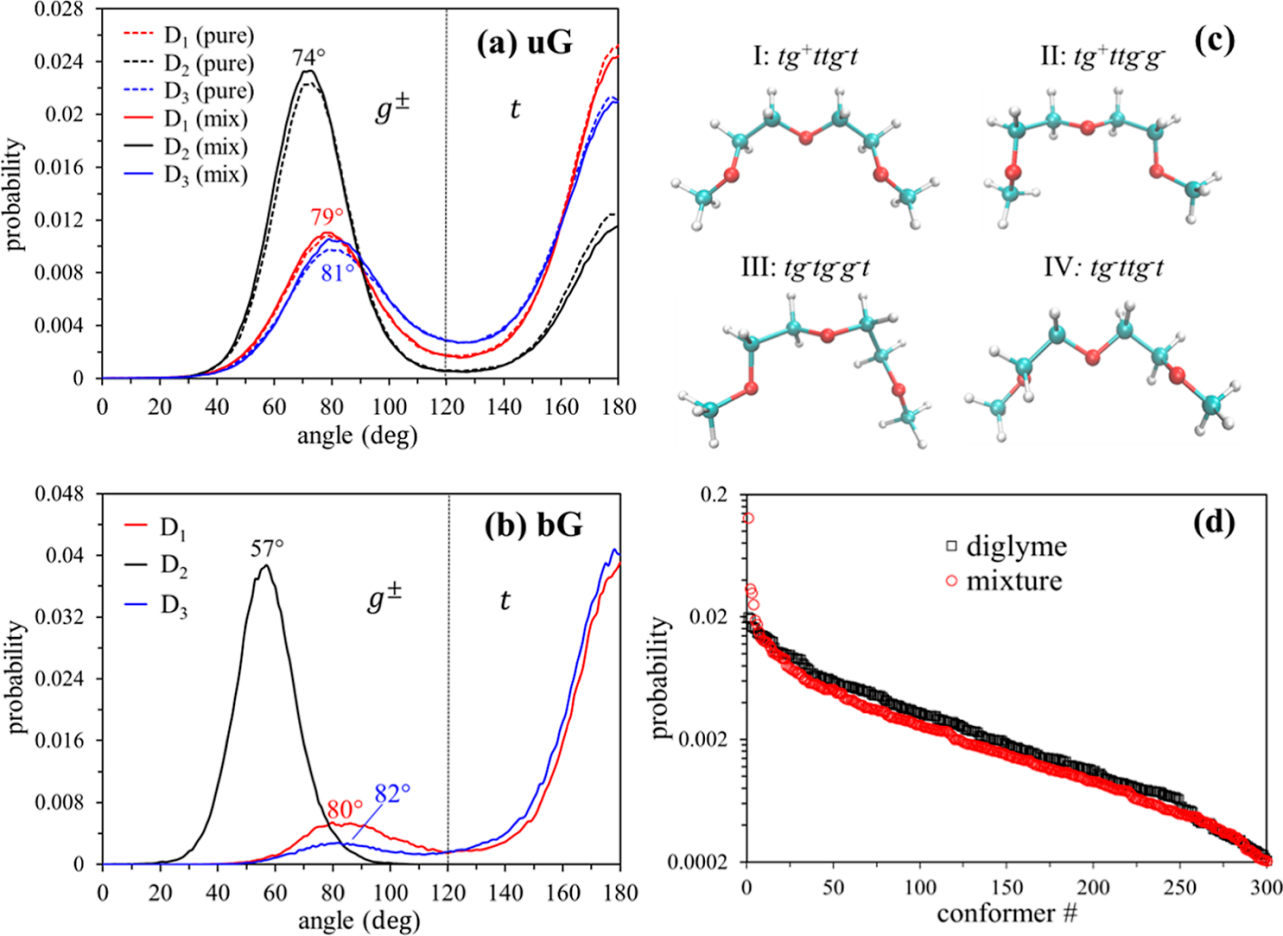
Dihedral angle distributions for (a) uG and (b) bG. *D*_1_, *D*_2_, and *D*_3_, respectively, denote the dihedral angles about the central bonds of CH_3_–O–CH_2_–CH_2_, O–CH_2_–CH_2_–O, and CH_2_–CH_2_–O–CH_2_. (c) Optimized gas phase structures of the four most populous bG conformers (excluding enantiomers, Table 5). Geometry optimization of bG was performed in the absence of Li^+^. (d) Distributions of diglyme conformations in pure diglyme (black) and in the diglyme + LiBF_4_ mixture (red). The distribution of uG conformations in the mixture is virtually identical with that in pure diglyme (black) and thus is not shown. In panels (a,b), the *x*-axis represents the absolute value of the dihedral angle as the distributions are symmetric about zero.

Because of the structural flexibility of diglyme, bound and unbound molecules are expected to have different conformations. To gain detailed insights, we analyzed probability distributions of the dihedral angles associated with the backbone atoms of bG and uG. The results for pure diglyme and the diglyme + LiBF_4_ mixture are shown in Figure 3a,b. *D*_1_, *D*_2_, and *D*_3_, respectively, denote the dihedral angles about the central bonds of CH_3_–O–CH_2_–CH_2_, the O–CH_2_–CH_2_–O, and CH_2_–CH_2_–O–CH_2_ segments. The trans (or anti) conformation, defined as having a dihedral angle in the range of −120 to −180° or 120 to 180°, is denoted as *t*, while gauche conformations, those having dihedral angles of 0–120° and −120–0° are represented as *g*^+^ and *g*^–^, respectively. Cis or eclipsed conformations, those with dihedral angles close to 0°, are not considered separately in this classification because their probability is extremely low, as indicated by Figure 3a,b. For simplicity, probabilities averaged over the positive and negative dihedral angles of the same magnitude are presented in Figure 3a,b. For instance, at 65°, we plot the average of *g*^+^ at 65° and *g*^–^ at −65°. Symmetry requires the probabilities be symmetric about 0°, and they are approximately equal in the present simulations.

With the above points in mind, we first consider the results for uG shown in Figure 3a. The probability distributions of all three dihedral angles are characterized by two maxima: one near 80° corresponding to *g*^±^, and one at 180° corresponding to *t*. Dihedral *D*_2_, in which the central bond is CH_2_–CH_2_, shows a preference for gauche conformations, whereas the trans conformation is preferred in dihedrals *D*_1_ and *D*_3_ where the central bond contains an O atom. It is noteworthy that none of these angles show a significant difference between neat diglyme and uG in the LiBF_4_ mixture, indicating that LiBF_4_ has little influence on the structure of uG. By contrast, the conformations of bG molecules (Figure 3b) are significantly different from those of uG. Most notable is that *D*_2_ occurs exclusively in gauche conformations with the maximum probability shifted to ±57°, revealing that its O atoms face generally the same direction, as needed for coordination to Li^+^ (cf. Figure 3c) (dihedral distributions very similar to those in Figure 3b were also obtained in classical MD simulations of Li^+^–triglyme systems^32,35^). As a result, the backbone structure of bG is generally less open than that of uG. This change in *D*_2_ is accompanied (or facilitated) by a considerable increase in the prevalence of trans conformations in the other two dihedral angles. In bG, 80% of the *D*_1_ and 90% of the *D*_3_ angles are trans, compared to 57% trans in neat diglyme and 56% for uG in the mixture for both of these angles. These differences in dihedral angle distributions imply that bG is more compact and less flexible than uG, as suggested by the depictions of the high-probability structures in Figure 3c. The radii of gyration (*R*_g_) listed in Table 4 provide a quantitative measure of this conformational preference. Both the average and standard deviation of *R*_g_ are considerably smaller for bG than those for uG.

**Table 4:** Radii of Gyration (*R*_g_) of Diglyme Molecules in Pure Diglyme and in the LiBF_4_ + Diglyme Mixture^a^

	*R*_g_ (Å)
uG (pure)	2.4 ± 0.24
uG (mix.)	2.4 ± 0.24
bG (mix.)	1.95 ± 0.15

a *R*_g_ values are averages based on the locations of oxygen atoms; ± vales are standard deviations.

To gain insights into the overall conformations of diglyme in these systems, we sorted molecules into conformers based on the definitions of *t*, *g*^+^, and *g*^–^ given above and ranked them according to their relative probability of occurrence. Before the conformer distributions so obtained are discussed, two points are worth mentioning. First, dihedral sequences *abcdef* and *fedcba*, e.g., *tg*^+^*ttg*^–^*g*^–^ and *g*^–^*g*^–^*ttg*^+^*t*, denote the same conformer. Despite this equivalence, the number of possible conformations for a diglyme molecule is still large, 378 to be exact (see notes on Backbone Conformations in the Supporting Information). Second, most conformers (to be specific, 364 conformers) come in enantiomeric pairs, for example, *tg*^+^*ttg*^–^*g*^–^ and *tg*^–^*ttg*^+^*g*^+^. In an achiral environment at equilibrium, the populations of two such enantiomers should be the same; however, incomplete sampling in an MD simulation may cause deviations from this equality. In the present simulations, we observe only a near equality in the populations of enantiomeric pairs, and we treat them as separate conformers.

We first consider the distribution of pure diglyme conformers, shown as black squares in Figure 3d. Although fewer than half of 378 possible conformations account for ∼90% of the population, the distribution is still quite broad and is such that 25 conformers occur with probabilities ≥1.0%, and no conformers occur with >2.0% probability (Table S1). The three most populous conformations were found to be *tttg*^–^*g*^+^*t* (2.0%), *tg*^–^*ttg*^–^*g*^+^ (1.9%), and *tg*^+^*tg*^–^*g*^+^*t* (1.7%). We note that there is little correspondence between the very broad distribution of conformers found here and the much more limited set of conformers predicted by single-molecule ab initio calculations^78,79^ to be present in neat diglyme.

By contrast, in the mixture, the occurrence of four conformer/enantiomer structures, *tg*^+^*ttg*^–^*t*, *tg*^+^*ttg*^–^*g*^–^/*tg*^–^*ttg*^+^*g*^+^, *tg*^–^*tg*^–^*g*^–^*t*/*tg*^+^*tg*^+^*g*^+^*t*, and *tg*^–^*ttg*^–^*t*/*tg*^+^*ttg*^+^*t*, is markedly enhanced compared to pure diglyme, as shown in Figure 3d and Table 5. Hereafter, these conformers will be denoted as conformer I, conformers II/II*, conformers III/III*, and conformers IV/IV*, respectively, where * indicates a mirror image. Most striking is that while conformer I (= conformer I*), *tg*^+^*ttg*^–^*t*, accounts for only 1% of the population in neat diglyme, more than 40% of bG is characterized by this conformation (Table 5). The optimized structures of these conformers in the gas phase, exhibited in Figure 3c, indicate that they are those that facilitate coordination of Li^+^. As indicated above, Table 5 shows that enantiomeric pairs are observed with slightly different populations due to incomplete AIMD sampling. Nevertheless, our results for bG conformations are in line with the gas-phase ab initio predictions that conformers I and III* are among the minimum energy structures of 1:1 and 2:1 diglyme–Li^+^ complexes.^39−42^ However, conformers II/II* and IV/IV* were not found in the gas-phase calculations. One possible explanation is the neglect of solvation in these calculations, which often plays an important role in determining the solution-phase structure. It is noteworthy that the conformation of diglyme in the diglyme–LiBF_4_ crystal is *tg*^+^*ttg*^–^*t* (conformer I) and the repeat unit of the PEO_3_–LiBF_4_ crystal is alternating sequences of *tg*^+^*ttg*^–^*t* and *tg*^–^*t*,^8^ lending further support to our finding that conformer I is the dominant conformation of bG in the solution phase.

**Table 5:** Probabilities of the Four Most Probable Enantiomeric Pairs in Pure Diglyme and the Diglyme + LiBF_4_ Mixture

conformer	I (= I*) *tg*^+^*ttg*^–^*t*	II + II* *tg*^+^*ttg*^–^*g*^–^ + *tg*^–^*ttg*^+^*g*^+^	III + III* *tg*^–^*tg*^–^*g*^–^*t* + *tg*^+^*tg*^+^*g*^+^*t*	IV + IV* *tg*^–^*ttg*^–^*t* + *tg*^+^*ttg*^+^*t*	I – IV total
uG (pure)	1.0%	0.4% + 0.5%	0.6% + 0.6%	1.4% + 1.0%	5.5%
bG (mix)	12.9%	3.4% + 2.5%	3.1% + 1.4%	1.9% + 1.7%	26.9%
bG (bG only)	44.0%	9.0% + 7.4%	10.0% + 2.4%	3.8% + 3.9%	80.5%

### 3.3 Far-IR Spectra

**Figure 4:**
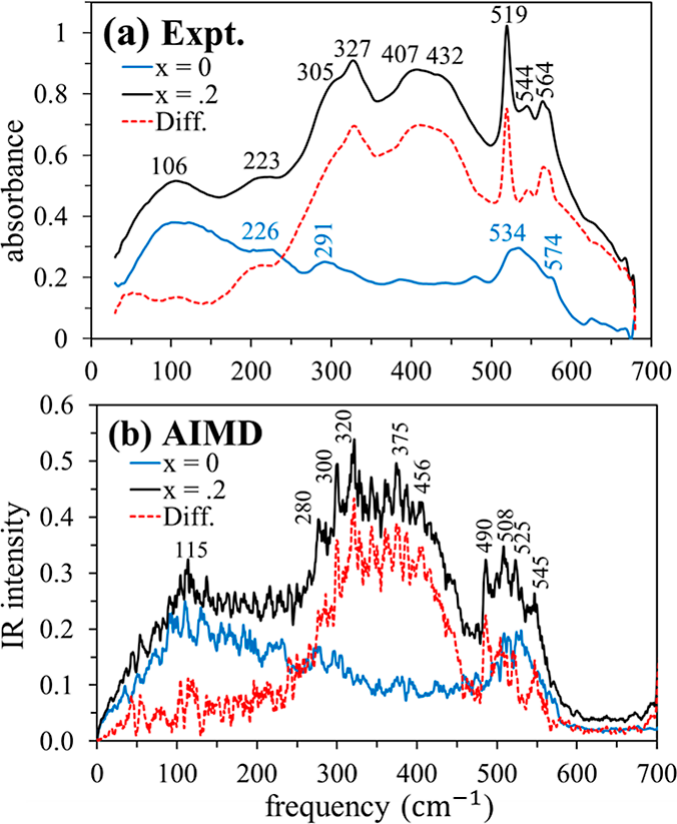
Far-IR spectra of pure diglyme (blue), the diglyme + LiBF_4_ mixture (black), and the difference spectrum (mixture - pure, red dashed): (a) experimental results; (b) AIMD results [*n*(ω)α(ω); units: 10^3^ cm^–1^].

Experimental and AIMD spectra of pure diglyme and the diglyme mixture (*x*_LiBF_4__ = 0.2) in the far-infrared region are exhibited in Figure 4a,b. To emphasize the influence of added LiBF_4_, the difference in the absorption intensity between the two systems is displayed in red. Before embarking on our analysis, we remind the reader that the experimental spectra were obtained at 294 K while simulations were performed at 350 K (Section 2). Despite this difference in temperature, computational spectra, though noisy due to limited AIMD statistics, nicely reproduce the main experimental observations. These include the double-peak nature of the new band in the 250–500 cm^–1^ region and the intensity enhancement with the development of multipeak structure in the 500–600 cm^–1^ region, though the latter feature is not as pronounced in AIMD as in experiment. Similar to the PC and IL mixtures with LiBF_4_ we studied previously,^21^ the current AIMD spectra are generally red-shifted with respect to the experiment, in this case by an average of ∼30 cm^–1^ for features between 200 and 700 cm^–1^.

**Figure 5:**
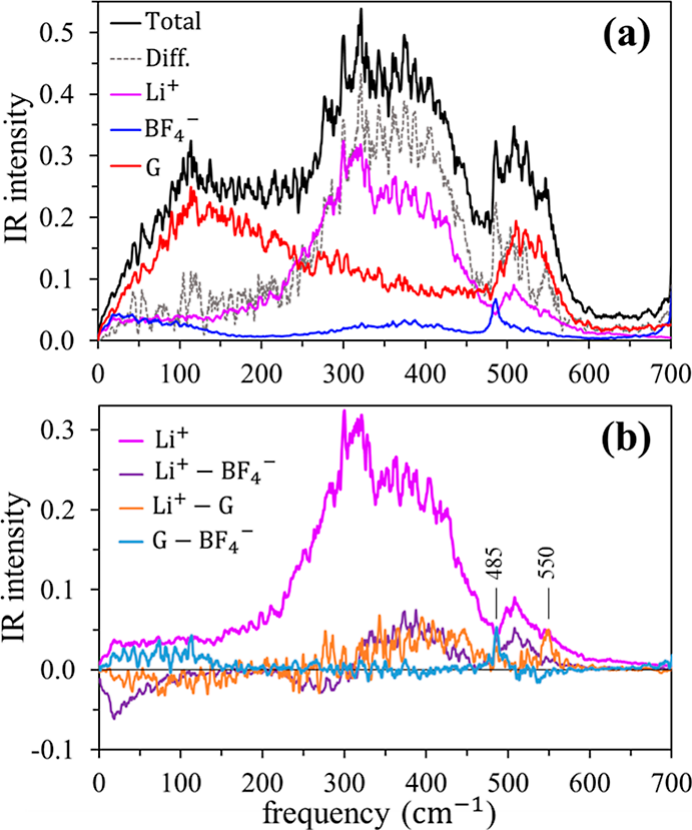
Decomposition of the AIMD far-IR spectrum of the diglyme + LiBF_4_ mixture. (a) Total and difference (mixture - pure diglyme) spectra compared to the contributions of the autocorrelation functions ⟨_A_(0)·_A_(*t*)⟩ of species A = Li^+^, BF_4_^–^, and diglyme (G). (b) Li^+^ self-contribution and contributions of various cross-correlations ⟨_A_(0)·_B_(*t*)⟩.

To understand the differing roles played by the components of the mixture, as shown in Figure 5, we decompose the spectrum into contributions of species auto- and cross-correlations, using eq 3 with MLWF-based electronic charges.^69−71^ Several aspects of these decompositions are worthy of note. First, the contribution from the autocorrelation of the Li^+^ current, ⟨_Li_(0)·_Li_(*t*)⟩, is indeed the primary contributor to development of the new band with a double-peak structure in the 250–500 cm^–1^ region; it accounts for more than 50% of the absorption intensity in this region (Figure 5a). As mentioned above, the motions responsible for this absorption are hindered translations, i.e., rattling vibrations of Li^+^ in its solvation cage, which, as we have seen, mainly consists of one bG and one or two BF_4_^–^ molecules (Table 3). Cross-correlations of the Li^+^ current with those of diglyme and BF_4_^–^ also make significant contributions to this Li-rattling band (Figure 5b), implying that motions of diglyme and BF_4_^–^ are coupled to Li^+^ vibrations (see below). Second, the intensity enhancement between 500 and 600 cm^–1^ is largely due to Li^+^ vibrations coupled with BF_4_^–^ motions, while combined motions of Li^+^ and diglyme are mainly responsible for the new peak near 550 cm^–1^ (564 cm^–1^ in the experiment). Third, the new peak at 485 cm^–1^ (519 cm^–1^ in the experiment) arises from BF_4_^–^ motions coupled with Li^+^ and diglyme. Finally, despite the coupling of diglyme motions with those of Li^+^ and BF_4_^–^, the spectrum resulting from diglyme alone in the mixture (red curve in Figure 5a) differs little from that of pure diglyme shown in Figure 4b. We note that these changes induced by LiBF_4_ in diglyme solution are quite similar to those in the LiBF_4_ + PC mixture.^21^

For additional insights, we analyzed the separate contributions of bG and uG to the IR spectrum. It should be pointed out that we have not observed any transitions between bG and uG, viz., dissociation of bG from Li^+^ solvates or its reverse process, binding of uG to Li^+^, in these simulations. This indicates that the lifetime of bG is longer than the ∼120 ps duration of the individual AIMD trajectories, an observation consistent with many earlier classical MD studies, where the residence times of Li^+^ in glymes and PEOs were found to be in the range of 1–1000 ns.^27−31,33,34,38^ These long lifetimes allow us to treat bG and uG as two different species and compute their autocorrelations and cross-correlations unambiguously within the simulation time scale. In addition, the fact that bG structures are relatively rigid enables analysis of its GNMs.^47,48^

**Figure 6:**
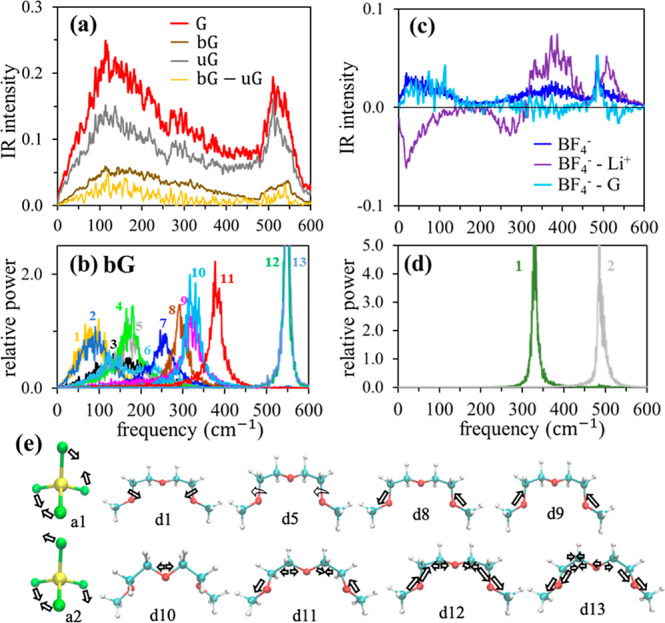
Spectral contributions and intramolecular vibrations of diglyme and BF_4_^–^ of the diglyme + LiBF_4_ mixture. Panels (a,c) show auto and cross contributions to the AIMD spectra from diglyme and BF_4_^–^, respectively. Panels (b,d) show power spectra of the GNMs of these two species, and panel (e) illustrates the motions associated with some of these modes. The straight and curved arrows in panel (e) represent bending and torsional motions, respectively.

Figure 6a shows the results of this further decomposition, and Figure 6b shows the power spectra of the GNMs of the most populous bG conformer, *tg*^+^*ttg*^–^*t* (I). Contributions of BF_4_^–^ and its two GNM spectra are shown for comparison in Figure 6c,d, and the motions associated with some of these GNMs are depicted in Figure 6e. (GNM spectra of conformers II and III are shown in Figure S3, and depictions of motions of all GNMs of bG conformers I–III for *v̅* ≤ 1100 cm^–1^ are provided in Figures S4–S6. In a given spectral region, vibrational characteristics are similar across conformers I–III.) The contribution of uG to the far-IR spectrum is significantly greater than that of bG, approximately in proportion to the 2.2:1 relative populations of uG/bG in the mixture. The shape of the uG contribution to the spectrum is nearly identical to that of pure diglyme, indicating that LiBF_4_ has little influence on the uG spectrum. This is expected because the dihedral angle distributions and conformer distributions of uG are essentially the same as those of pure glyme (Figure 3a,d). The uG and bG spectral line shapes are also similar, except in the 500–550 cm^–1^ region. The broad and structureless character of the absorption below 400 cm^–1^ is ascribed to the presence of a large number of different diglyme conformers (Figure 3d and Table S1). Judging from the GNMs of bG, each of these conformers has roughly 10 low-frequency modes, which entail flexing of the backbone via torsional and/or bending motions (Figures 6b,e and S3–S6). They can couple to similar modes of other diglyme conformers as well as to hindered translational and rotational motions. This leads to a myriad of nonlocal motions with a broad distribution of frequencies for *v̅* ≲ 400 cm^–1^. In the 500–550 cm^–1^ region, the bG band becomes skewed toward higher frequencies compared to the uG band, such that the maximum in this region shifts from ∼510 cm^–1^ in uG to ∼550 cm^–1^ in bG. These changes are attributed mainly to the high probability of occurrence of conformer I (*tg*^+^*ttg*^–^*t*) in bG compared to the uG case (Table 5). In view of the power spectra of the conformer I bending modes d12 and d13, which fall within the 500–600 cm^–1^ region, and the absence of GNMs between ∼400 and 500 cm^–1^ (Figure 6b), conformer I will contribute a distinct peak centered at around 550 cm^–1^. The relative intensity of this peak is expected to be higher in the bG spectrum than that in the uG because of the large population of conformer I in bG, skewing the bG band to 550 cm^–1^ with respect to the uG band. Based on these results, we assign the 545 cm^–1^ structure in the AIMD spectrum (564 cm^–1^ in the experiment) primarily to d12 and d13 of bG (and related bending motions of uG), coupled with Li^+^ vibrations.

Turning to BF_4_^–^ in Figure 6c,d, we notice that the power spectrum of its two degenerate symmetric bending (a1) modes overlaps significantly with the IR intensity resulting from its current autocorrelation in the 250–450 cm^–1^ region, suggesting the participation of these modes in IR absorption there. Since fully symmetric a1 vibrations (Figure 6e) are IR-inactive, we conclude that BF_4_^–^ contributes to the Li-rattling band through bending motions of broken a1 symmetry (induced by coordination with Li^+^) combined with its center-of-mass vibrations with respect to Li^+^ (see Figure S8). This situation is very similar to what we previously observed in PC and IL mixtures with LiBF_4_; specifically, intramolecular vibrations of BF_4_^–^ and solvent molecules in and near the Li^+^ coordination shell, as well as vibrations of the coordination shell itself, are all coupled to Li^+^ motions in the 250–500 cm^–1^ region. The power spectrum of the three degenerate antisymmetric bending (a2) modes of BF_4_^–^ (Figure 6d) peak near 485 cm^–1^, nearly coinciding with IR spectral contributions arising from the BF_4_^–^ current autocorrelation as well as cross-correlations between BF_4_^–^–diglyme and between BF_4_^–^–Li^+^. This coincidence indicates that the new peak at 485 cm^–1^ (519 cm^–1^ in the experiment) in the mixture is due to a2 vibrations of BF_4_^–^, combined with Li^+^ and diglyme vibrations.

In the low-frequency region, *v̅* ≲ 150 cm^–1^, the far-IR spectrum of the mixture arises mainly from diglyme. GNMs d1–d5 of bG (Figures 6b,e and S3–S6) suggest that relevant diglyme motions here are backbone torsions (d2–d5) combined with overall bending (d1). This band is largely unaffected by LiBF_4_, except that its intensity is slightly enhanced by contributions from the added lithium salt. For *v̅* ≲ 120 cm^–1^, the Li^+^ and BF_4_^–^ currents are anticorrelated, whereas their center-of-mass velocities are positively correlated (Figure S8), indicating that Li^+^ and its coordinated BF_4_^–^ (and bG) translate together in this frequency range. Concerted Li^+^ coordination sphere motions of this sort were also found below 150 cm^–1^ in the PC and IL mixtures.^21^

### 3.4 Mid-IR Spectra

**Figure 7:**
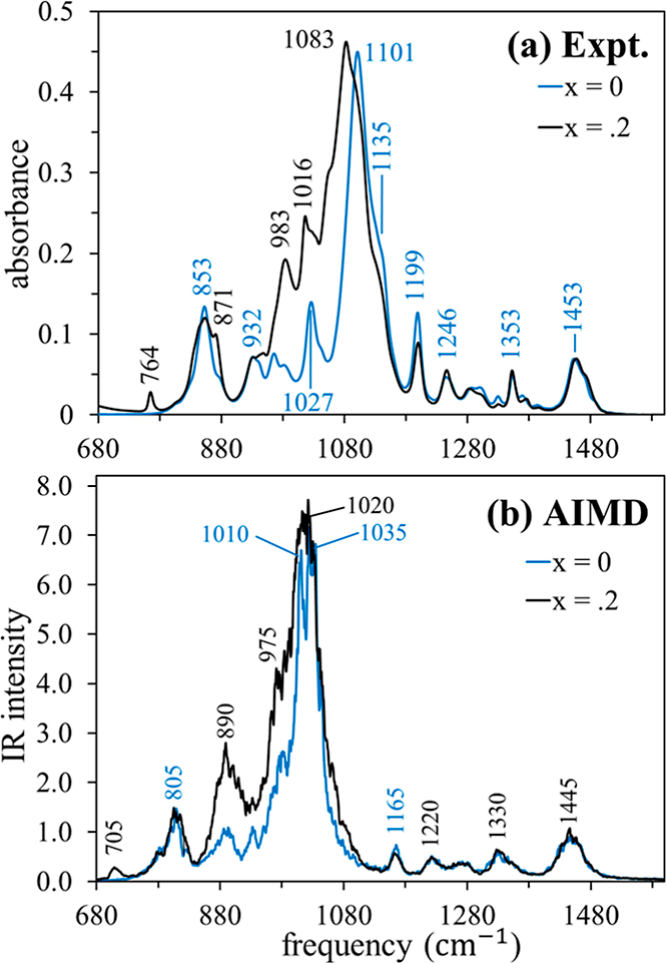
(a) Experimental and (b) AIMD spectra of diglyme and diglyme + LiBF_4_ in the mid-infrared region. The spectra in panel (b) are products *n*(ω)α(ω) with intensity units of 10^3^ cm^–1^.

**Figure 8:**
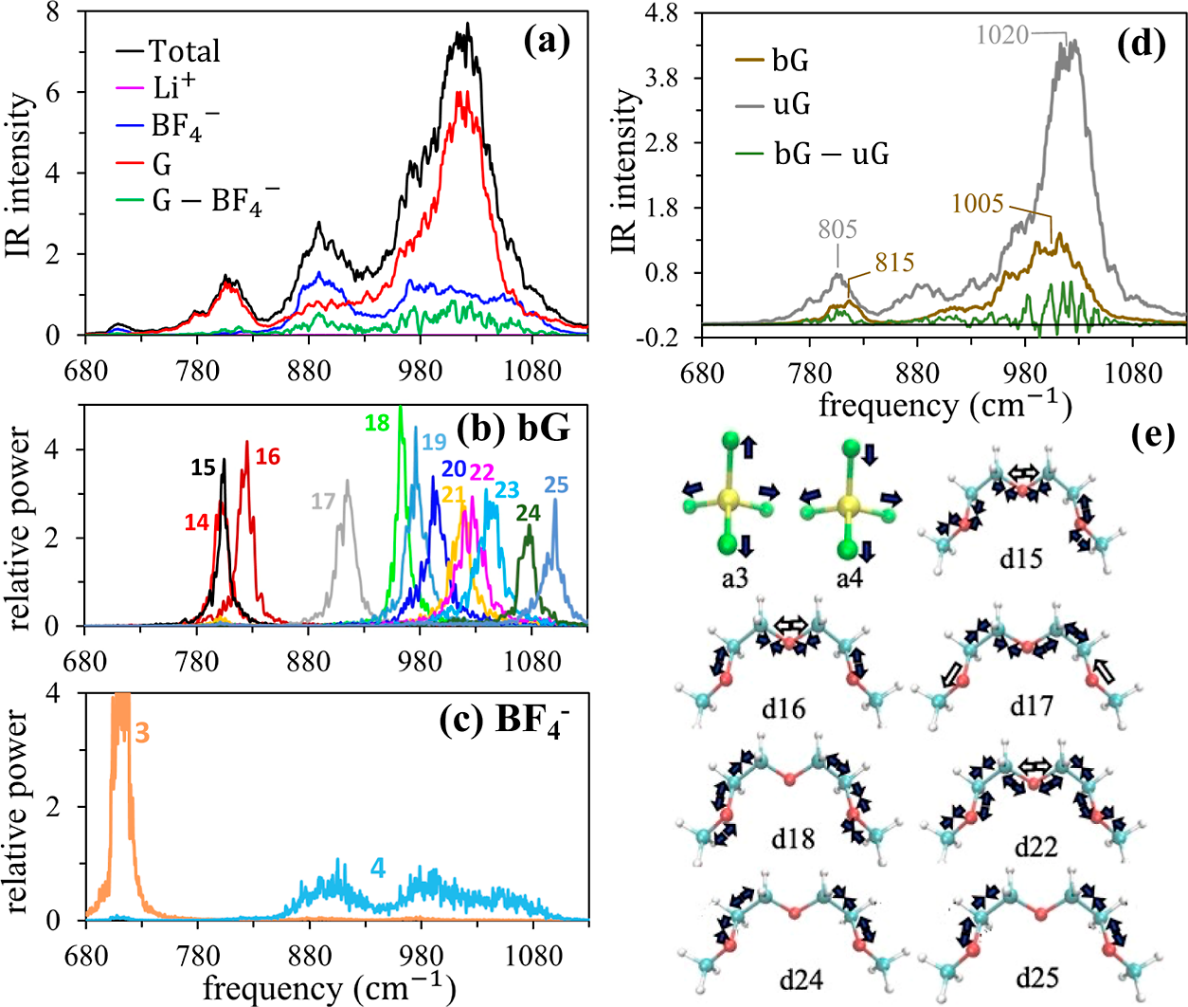
(a) Total AIMD spectrum and the contributions of the autocorrelation functions ⟨_A_(0)·_A_(*t*)⟩ from species A = Li^+^, BF_4_^–^, and diglyme (G), as well as the cross-correlation ⟨_G_(0)·_BF_4__(*t*)⟩. (b,c) Power spectra of GNMs of bG and BF_4_^–^. (d) Contributions of bG and uG to the spectrum. (e) Representations of some GNMs. Open arrows represent bending and closed arrows stretching motions. Though not shown as arrows, C–H bending motions are coupled with backbone stretching vibrations in diglyme GNMs due to intermolecular interactions.

We now turn to the mid-IR spectra of pure diglyme and the LiBF_4_ + diglyme mixture in the fingerprint region (Figures 7a,b and 8a–e) (the corresponding experimental spectrum of the LiTfO + diglyme mixture is presented in Figure S2a). As seen in Figure 7, both experiment and simulation show that the main spectral changes caused by the addition of LiBF_4_ are confined to the 700–1150 cm^–1^ region. In all other regions, only modest differences between the mixture and neat diglyme spectra—mainly in their peak intensities—are found, suggesting limited influence of Li^+^ or BF_4_^–^. Similar to prior observations of Li^+^ in the PC and the IL mixtures, contributions from time correlation functions involving Li^+^ currents are negligible throughout the entire mid-IR region, disclosing that Li^+^ dynamics do not play any role in the mid-IR spectrum. As in the far-IR region, simulated features are red-shifted compared to the experiment, here by an average of ∼70 cm^–1^. As seen in Figure 7, the intense absorption band in pure diglyme, which consists of a main peak at 1101 cm^–1^ (1035 cm^–1^ in AIMD) with a secondary peak at 1027 cm^–1^ and a shoulder around 1135 cm^–1^, exhibits a pronounced intensity enhancement and a significant broadening on its red side in the mixture, accompanied by a red-shift of its main peak by ∼20 cm^–1^ (Figure 7a). (In contrast to the effect of LiBF_4_, this band is not significantly influenced by the addition of LiTfO to pure diglyme. See Figure S2a). These spectral changes, very similar to the PC mixture case,^21^ are reasonably captured by AIMD (Figure 7b).

Figure 8 shows a decomposition analysis of this region. Figure 8a reveals that (i) the primary band indeed derives mostly from diglyme in the mixture and (ii) motions of BF_4_^–^ coupled with diglyme confer a double-peaked character to the spectrum (blue and green lines in Figure 8a), viz., a peak around 890 cm^–1^ and a broad structure between 950 and 1100 cm^–1^, resulting in the intensity enhancement and broadening observed in the experiment. The power spectrum of the three degenerate antisymmetric stretching (a4) modes of BF_4_^–^ (Figure 8c) coincides with the spectral contribution made by the BF_4_^–^ current autocorrelation (blue line in Figure 8a), indicating that B–F stretching vibrations are responsible for these changes in the mixture. The double-peak character of the spectrum noted above is attributed to coordination of Li^+^ by BF_4_^–^, which results in two different types of F atoms, those directly coordinating Li^+^ and the remainder. Since the B–F bonds of the former are weakened compared to those of the latter, the lower and higher frequency structures in the BF_4_^–^ spectrum are assigned to stretching vibrations of coordinating and noncoordinating F, respectively. Interestingly, the power spectrum of a4 is much broader than those of the other GNMs, a1–a3, of BF_4_^–^. The difference is ascribed to strong coupling of a4 to different vibrational motions of diglyme. Based on the GNM results, there are roughly nine different modes, various backbone stretching vibrations combined with C–H bending motions, of bG in the 850–1100 cm^–1^ region. These modes, as well as the corresponding vibrations of uG in this frequency region, interact with a4, resulting in significant broadening of the a4 power spectrum. Furthermore, the distribution of these diglyme modes is such that roughly 1–2 (d17 and d18 of bG) and 7–8 (d18–d25 of bG) of them are respectively available for coupling to coordinating and noncoordinating F atoms of BF_4_^–^, which explains the much greater breadth of the high-frequency portion of the a4 power spectrum (a broad GNM spectrum of double-peak character was obtained for a4 also in the PC mixture). Based on these observations, we conclude that the marked intensity enhancement of the peak at 890 cm^–1^ (983 cm^–1^ in the experiment) is due to B–F stretching vibrations of Li^+^-coordinating F, while those of noncoordinating F atoms are the major contributors to the intensity enhancement and broadening of the band in the ∼950–1000 cm^–1^ region (∼1050–1100 cm^–1^ in the experiment).

AIMD also predicts a red-shift, albeit very small, of the center of the main peak from 1010/1035 to 1020 cm^–1^ (Figure 7b) This shift is attributed to two factors: downshift of the bG peak to 1005 cm^–1^ with respect to the uG peak at 1020 cm^–1^ (Figure 8d) and the decreasing intensity of the BF_4_^–^ spectrum with *v̅* (blue line in Figure 8a) in this frequency region (i.e., BF_4_^–^ contributes to, and enhances the intensity of, the lower frequency side of the band more than the higher frequency side). According to GNM analysis, the main motions of bG in this frequency region are stretching vibrations of the diglyme backbone coupled with various C–H bending motions (Figures 8e and S4–S6). Because the diglyme C–O bonds become weakened when their O atoms coordinate to Li^+^, C–O stretching vibrations involving these coordinating O atoms shift to lower frequencies. Gas-phase ab initio calculations also predict that binding to Li^+^ lowers CO stretching frequencies of various glyme molecules in the 1100–1200 cm^–1^ region.^43−45^

Other spectral changes in the fingerprint region (Figure 7) include the appearance of a new peak at 764 cm^–1^ (705 cm^–1^ in AIMD) and enhancement of the shoulder structure at 871 cm^–1^ on the blue side of the 853 cm^–1^ band (805 cm^–1^ in AIMD). The former is assigned to the symmetry-broken a3 stretching vibration of BF_4_^–^ as the a3 power spectrum in Figure 8c fully overlaps with this new peak. The shoulder at 871 cm^–1^ in the experiment is ascribed to the bG peak at 815 cm^–1^ in AIMD, which is blue-shifted with respect to the uG peak at 805 cm^–1^ (Figure 8d).

**Figure 9:**
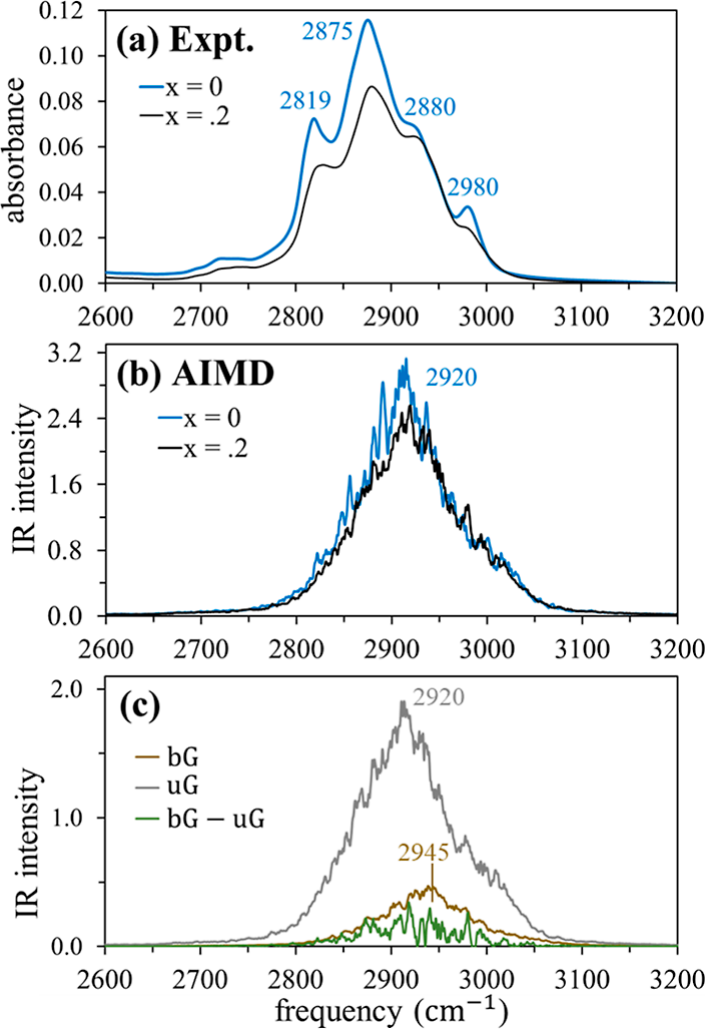
Spectra in the CH stretching region of diglyme and the diglyme + LiBF_4_ mixture: (a) experimental results; (b) AIMD results [*n*(ω)α(ω); units: 10^3^ cm^–1^]; and (c) spectral contributions of bG, uG and their cross correlation.

IR spectra in the C–H stretching region, 2700–3300 cm^–1^, are presented in Figure 9a–c. Spectral influence of LiBF_4_ is minimal in this region; the C–H stretching band shifts to higher frequencies by ∼5 cm^–1^, and the definition of the subpeaks and shoulders is reduced in the mixture. Interestingly, the IR spectrum of the LiTfO mixture in this frequency region (Figure S2b) is essentially identical with that of the LiBF_4_ mixture. This suggests that the distributions of bG conformations in these two lithium salt solutions are similar. We notice that the AIMD spectra are considerably less structured than their experimental counterparts. In addition, the former are blue-shifted with respect to the latter, in contrast to the far-IR and fingerprint regions. Similar differences between AIMD and experimental CH-stretching spectra were noted in our previous study of the PC and IL mixtures.^21^ AIMD nonetheless reproduces the small blue-shift observed in the mixture spectrum compared with neat diglyme. This shift is attributed to the presence of the bG peak at 2945 cm^–1^ in the mixture, which is 25 cm^–1^ higher than the corresponding uG peak (Figure 9c).

**Figure 10:**
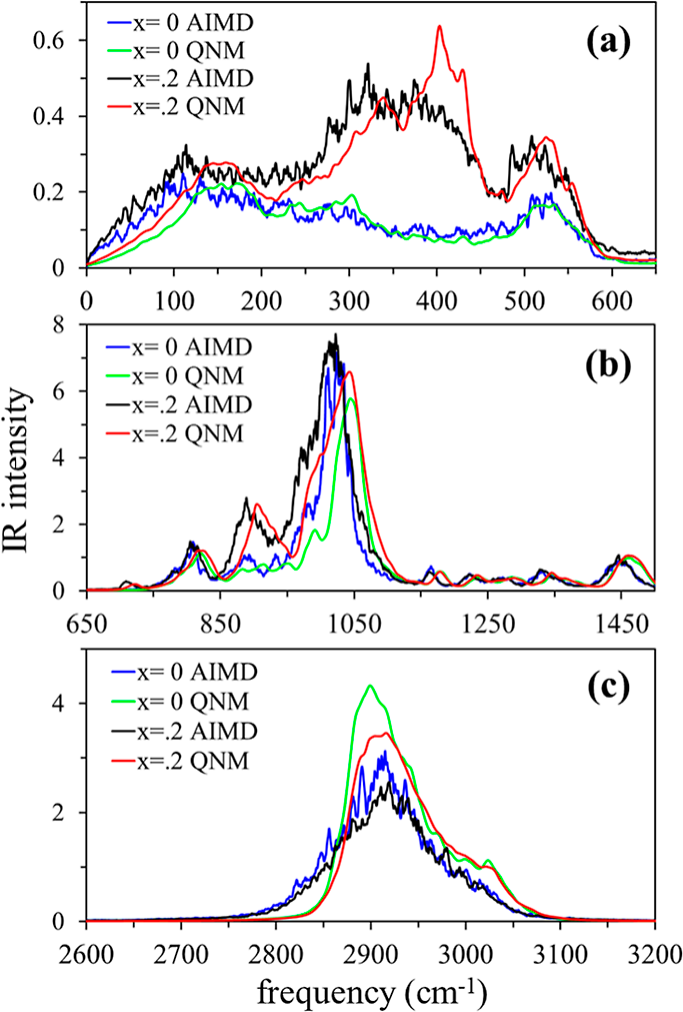
Comparison of AIMD and QNM spectra in the far-IR (a), fingerprint region (b), and CH-stretching region (c). The intensity of the QNM spectrum is normalized to have the same total area as the AIMD spectrum.

Finally, we consider IR spectra determined with the QNM method. QNM predictions for both the pure and mixture systems are compared to the AIMD spectra, as shown in Figure 10. Aside from a blue-shift of the QNM peaks relative to AIMD peaks, the two calculations yield similar spectra, as noted in our prior study.^21^ As pointed out there, this similarity indicates the absence of dynamic effects that substantially alter the inhomogeneous distribution of frequencies set by the structural heterogeneity of these systems. The blue-shift and apparent compression of the spectra toward higher frequencies observed with QNM calculations are expected because quenching into minimum energy structures reduces anharmonic contributions, thereby maximizing the force constants and frequencies. The largest deviations between the two calculations occur for large-amplitude vibrations on potential energy surfaces of high anharmonicity, such as found in the Li^+^ rattling region between 250 and 500 cm^–1^. Nonetheless, the main features of the AIMD spectra are reproduced reasonably well by QNM calculations. In this context, the diglyme systems studied here offer another example that supports our earlier proposal for IR calculations,^21^ viz., classical MD simulations followed by energy optimization and normal-mode analysis via quantum chemistry. In principle, this will allow for both better sampling and higher-level ab initio methods than those employed here, thereby providing potentially more accurate IR spectra, especially in the mid-IR region.

## 4 Conclusions

In this article, we have studied IR spectra of mixtures of diglyme and lithium salts, extending our recent work on related IL and PC solutions.^21^ We considered solutions of two different salts in experiment, LiBF_4_ and LiTfO, which show very similar far-IR spectra. Computational analysis of the former system was performed via ab initio MD simulations to gain in-depth understanding of the experimental results, especially the influence of added lithium salts on the IR spectra of diglyme solutions. The structure and composition of lithium solvates were examined and dynamics of individual components of the mixture and their correlated motions in these solvation environments were related to key spectral features.

One of our major findings was that hindered translations of Li^+^ ions in their solvation cages lead to the development of a new broad absorption band in the 250–500 cm^–1^ frequency region. Decomposition analysis of the LiBF_4_ + diglyme mixture shows that these rattling motions of solvated Li^+^ are strongly correlated with intramolecular vibrations as well as hindered translations of surrounding ions/molecules. According to GNM analysis, the symmetric bending of BF_4_^–^ and the backbone bending of diglyme are likely intramolecular vibrations that couple to Li^+^ dynamics. Structural analysis indicates that Li^+^ solvation is characterized by diverse coordination environments, suggesting a wide range of differing Li–solvent interactions, which explains the broad nature of the Li band. At low frequencies, *v̅* ≲ 120 cm^–1^, Li^+^ current is anticorrelated with the currents of BF_4_^–^ ions (and bG molecules), indicating that Li^+^ and its solvation cage generally translate together in this region.

Another important consequence of adding LiBF_4_ to pure diglyme is the broadening and intensity enhancement of the red side of the mid-IR band in the 900–1150 cm^–1^ region. These changes derive primarily from antisymmetric B–F stretching vibrations of BF_4_^–^—which are directly influenced by coordination of F to Li^+^—coupled with backbone stretching and C–H bending vibrations of diglyme. Also observed was a small red-shift of this band in the mixture, which was attributed mainly to coordination of oxygen atoms of diglyme to Li^+^ and resulting downshift of its C–O stretching frequencies compared to neat diglyme. The appearance of a weak but distinctive peak at 871 cm^–1^ is assigned to the symmetric stretching vibration of BF_4_^–^, whose symmetry is broken by the coordination with Li^+^.

As expected, the addition of LiBF_4_ was found to exert a strong influence on the diglyme structure. Backbone conformations of diglyme that facilitate its coordination with Li^+^ show a marked increase in the population in the mixture. While two of the most populous conformations of bG obtained in our simulations were previously found in ab initio calculations of 1:1 and 2:1 diglyme–Li^+^ complexes in the gas phase, several other conformations with a high probability of occurrence in AIMD were not. Though the level of ab initio theory employed in the present work is different from that in previous gas-phase studies, our finding nonetheless implies that solvation plays a critical role in the Li^+^ solvate structure, especially when coordinating molecules are as flexible as glymes.

The present study, together with our prior report on PC and IL mixtures with LiBF_4_,^21^ illustrates the utility of AIMD simulations, complemented by spectral decomposition using MLWFs and analysis of GNMs, for interpreting the IR spectra of electrolyte solutions in terms of solution structure and dynamics. For example, it can provide information about the contributions of motions of all component species as well as the coupling between these motions, thereby revealing the importance of collective and correlated molecular motions throughout the spectrum. Though not considered here, further decomposition of the spectra into contributions from electronic and nuclear charges of component species can shed light on the interaction-induced effect on the IR spectra.^21^ Therefore, despite its high computational cost, it would be worthwhile in the future to apply this approach to other important solution systems to understand their IR and related properties.

## Supporting Information Available

Diglyme backbone conformations; conformers with probability of occurrence ≥1.0%; experimental far-IR spectra of LiBF_4_ in diglyme, EmimBF_4_, and PC; comparison of experimental spectra of neat diglyme and mixtures of diglyme with LiBF_4_ and LiTfO; power spectra and schematic representations of low-frequency and mid-IR GNMs of bG conformers I–III in the LiBF_4_ mixture; contributions of current cross-correlations between Li^+^, BF_4_^–^, and bG to the IR spectra; and power spectra of the cross-correlation functions of center-of-mass velocities (PDF)
