# Supplementary material for: Infrared Spectroscopy of Li+ Solvation in Diglyme: Ab Initio Molecular Dynamics and Experiment
Source: J Phys Chem B. 2023 Oct 11;127(42):9191–203. doi: 10.1021/acs.jpcb.3c05612 (PMC10614183; doi:10.1021/acs.jpcb.3c05612)
Supplement: Supplementary file 1 — jp3c05612_si_001.pdf [file jp3c05612_si_001.pdf]

Supporting Information for

Infrared Spectroscopy of Li^+ Solvation in Diglyme: Ab Initio Molecular Dynamics and Experiment

Fangyong Yan,† Kallol Mukherjee,‡ Mark Maroncelli,‡ and Hyung J. Kim†

†Department of Chemistry, Carnegie Mellon University, Pittsburgh, PA 15213

‡Department of Chemistry, The Pennsylvania State University, University Park, PA 16802

Notes on Backbone Conformations S2

Table S1. Conformers with the probability of occurrence ≥ 1.0 % in pure diglyme and the diglyme + $LiBF_4$ mixture (x_{LiBF_4} = 0.2).. S4

Figure S1. Comparison of experimental far-IR spectra of 20 mol% solutions of $LiBF_4$ in diglyme, 1-ethyl-3-methylimidazolium tetrafluoroborate, and propylene carbonate S5

Figure S2. Comparison of experimental spectra of neat diglyme and 20 mol% mixtures of diglyme with $LiBF_4$ and LiTfO ($LiCF_3SO_4$).. S6

Figure S3. Power spectra of the low-frequency generalized modes of the three most populous bound diglyme conformers in the $LiBF_4$ mixture. S7

Figure S4. Schematic representations of the generalized normal modes of conformer I (*tg⁺ttg⁻t*) of bound diglyme S8

Figure S5. Schematic representations of the generalized normal modes of conformer II (*tg⁺ttg⁻g⁻*) of bound diglyme. S9

Figure S6. Schematic representations of the generalized normal modes of conformer III (*tg⁻tg⁻g⁻t*) of bound diglyme. S10

Figure S7. Power spectra of the mid-IR generalized modes of the three most populous bound diglyme conformers in the $LiBF_4$ mixture. S11

Figure S8. (a) Contributions of current cross-correlations, $\langle \vec{J}_A(0) \cdot \vec{J}_B(t) \rangle$, between Li^+, BF_4^-, and bound diglyme to the IR spectra. (b) Power spectra of the cross-correlation functions of center-of-mass velocities, $\langle \vec{v}_A(0) \cdot \vec{v}_B(t) \rangle$, of these same species S12

Notes on Backbone Conformations

For perspective on the diglyme conformations observed in simulation we sought to know the total number of conformers available, at least at the crudest level of approximation. Because we failed to find a method for calculating this number in the literature, we provide the following derivation. We consider dihedral conformations of a polymer backbone consisting of n independent dihedral angles, each of which can assume one of m different conformations. We represent the overall conformation of the polymer backbone as an ordered sequence, $a_1a_2a_3 \cdots a_n$, where a_j is the conformation of the j-th dihedral angle D_j along the backbone. The diglyme system we studied (Figure 3) corresponds to the case $n = 6$ (D_1-D_6) and $m = 3$ (t, g^+ and g^-). Since there are m possibilities for each individual dihedral angle, there are m^n different ordered sequences. The collection of these m^n sequences is referred to as the sequence set. By symmetry, many pairs among the sequence set represent the same conformer; to find the actual number of distinguishable conformers such pairings must be removed. To do so we proceed as follows. First, we consider only polymers like polyethylene oxide whose two termini are equivalent. In such polymers any pair of a_j sequences that are in reverse order of each other (i.e., $abc \cdots fgh$ and $hgf \cdots cba$) represent the same backbone conformation. This equivalence means that some backbone conformations are doubly represented in the sequence set, once as an a_j sequence and once as its reverse. To account for this duplicity, we classify ordered a_j sequences into two groups, symmetric and asymmetric sequences. The former are sequences that remain unchanged when the order is reversed, for example tg^-ttg^-t (conformer IV in Figure 3c). Symmetric ordered sequences appear only once in the sequence set, and the number of different symmetric conformers, $S_m(n)$, is the same as the number of different symmetric sequences in the sequence set. Since symmetric sequences are completely specified by a_j's in the first half of the sequence, this number is given by

$$S_m(n) = m^{n/2} \quad \text{for even } n \tag{S1}$$

$$S_m(n) = m^{(n+1)/2} \text{ for odd } n. \tag{S2}$$

The asymmetric group comprises the remainder of sequences in the sequence set, i.e., those lacking symmetry under sequence reversal. For any given asymmetric sequence, its reverse sequence is also present in the sequence set, but it does not represent a distinct conformer. Because of this duplication, the number of distinct asymmetric conformations, $A_m(n)$, is one half the number of different asymmetric a_j sequences

$$A_m(n) = (m^n - S_m(n))/2. \tag{S3}$$

The total number of different distinguishable backbone conformations, $T_m(n)$, is then the sum of $A_m(n)$ and $S_m(n)$

$$T_m(n) = A_m(n) + S_m(n) = (m^n + S_m(n))/2 = m^{n/2}\,(m^{n/2} + 1)/2 \qquad \text{for even } n \tag{S4}$$

$$= m^{(n+1)/2}\,(m^{(n-1)/2} + 1)/2 \qquad \text{for odd } n \tag{S5}$$

For the diglyme case considered in the main article, $n = 6$ and $m = 3$, the total number of different conformations is $T_3(6) = 3^3\,(3^3 + 1)/2 = 378$.

Table S1. Conformers with the probability of occurrence ≥ 1.0 % in pure diglyme and the diglyme + $LiBF_4$ mixture ($x_{\mathrm{LiBF_4}}$ = 0.2).

Pure diglyme		Mixture	
Conformation	Probability (%)	Conformation	Probability [a] (%)
$tttg^-g^+t$	2.0	tg^+ttg^-t	12.9 (44.0)
$tg^-ttg^-g^+$	1.9	$tg^+ttg^-g^-$	3.4 (9.0)
$tg^+tg^-g^+t$	1.7	$tg^-tg^-g^-t$	3.1 (10.0)
$tg^+ttg^-g^+$	1.6	$tg^-ttg^+g^+$	2.5 (7.4)
$ttttg^+t$	1.6	tg^-ttg^-t	1.9 (3.8)
$ttttg^-t$	1.5	tg^+ttg^+t	1.7 (3.9)
$tttg^+g^-t$	1.5	$tg^+g^-tg^-t$	1.5 (0.9)
$tttg^+g^+t$	1.4	$tg^+tg^+g^+t$	1.4 (2.4)
$tg^+tg^+g^-t$	1.4	$tg^+tg^+g^-t$	1.3 (0.7)
tg^-ttg^-t	1.4	$ttttg^-t$	1.3 (0.1)
$tg^+g^-tg^-t$	1.4	$tg^-g^-tg^-g^-$	1.3 (3.4)
$tg^-ttg^+g^-$	1.3	$tg^+tg^-g^+t$	1.2 (--)
$tg^+g^-g^-g^-t$	1.3	$tg^-ttg^+g^-$	1.2 (0.01)
$g^+g^-ttg^+g^-$	1.3	$tg^-tg^+g^-t$	1.1 (--)
$tg^-g^+tg^+g^-$	1.3	$tg^-ttg^-g^-$	1.0 (2.0)
$tg^-g^+ttg^+$	1.1	$tttg^+g^-t$	1.0 (1.8)
$ttttg^+g^-$	1.0	$tg^-tg^-g^+g^+$	1.0 (1.8)
$tg^-tg^+g^-t$	1.0	$ttttg^+t$	1.0 (0.01)
$tg^+ttg^+g^+$	1.0	$tg^+ttg^+g^-$	1.0 (--)
tg^+ttg^-t	1.0		
$tg^+g^-tg^-g^+$	1.0		
ttg^-tg^+t	1.0		
$tg^+tg^-g^-t$	1.0		
$tg^+ttg^+g^-$	1.0		
tg^+ttg^+t	1.0		

[a] Relative probabilities of finding a conformer within the bG population are given in parentheses. (--) means that no occurrence of this conformer was observed in the bG distribution.

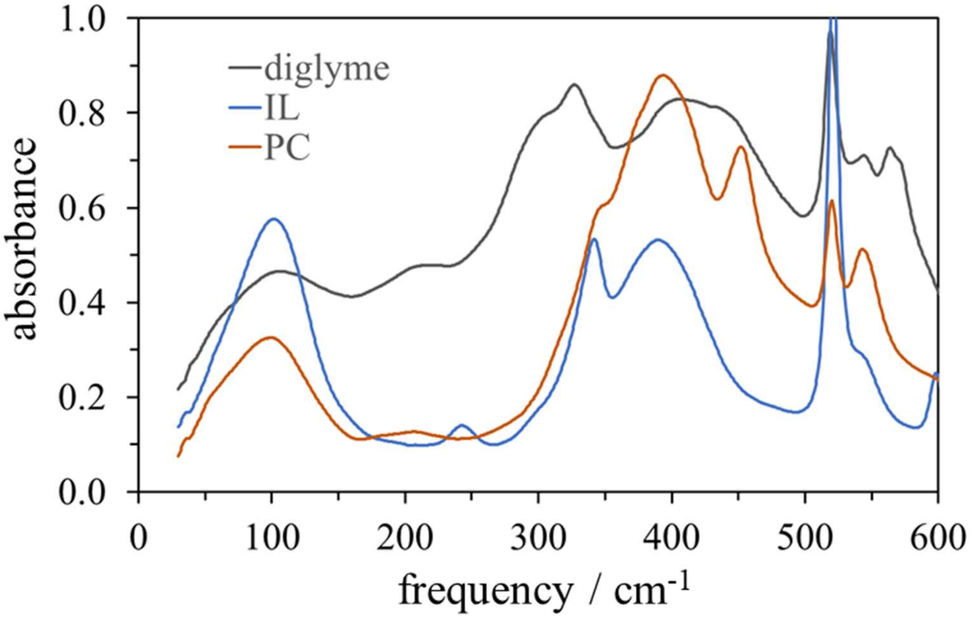

Figure S1. Comparison of experimental far-IR spectra of 20 mol% solutions of $LiBF_4$ in diglyme, 1-ethyl-3-methylimidazolium tetrafluoroborate ("IL" or $EmimBF_4$), and propylene carbonate ("PC"). All spectra were recorded in transmission mode on 6 μm thick samples. No solvent subtraction was performed.

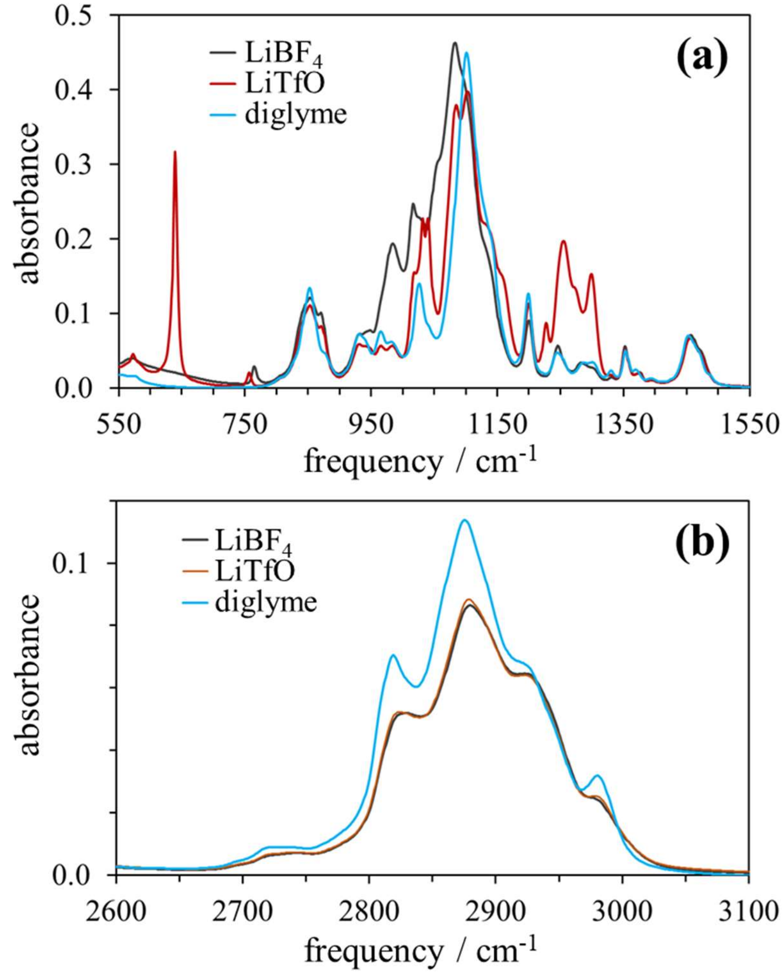

Figure S2. Comparison of experimental spectra of neat diglyme and 20 mol% mixtures of diglyme with $LiBF_4$ and LiTfO ($LiCF_3SO_4$). Spectra were recorded in attenuated total reflection mode using a diamond ATR crystal.

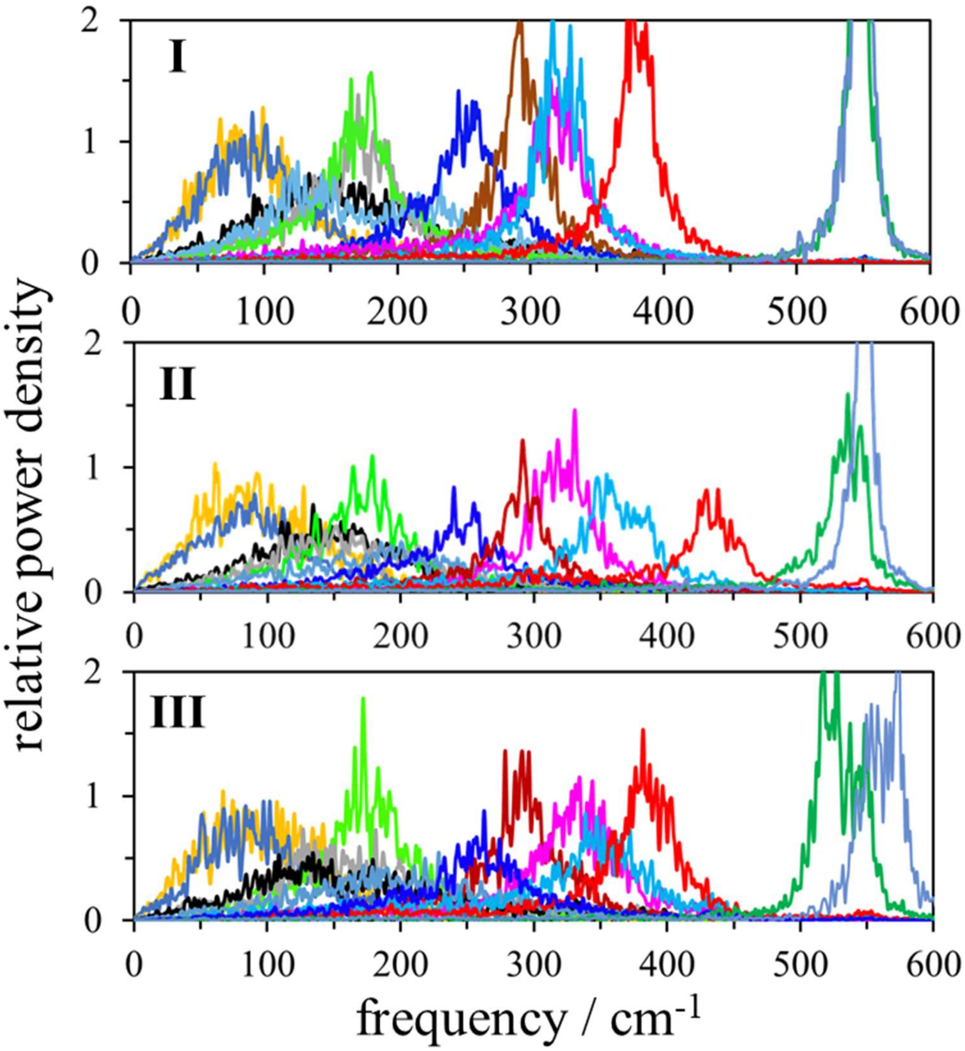

Figure S3. Power spectra of the low-frequency generalized modes of the three most populous bound diglyme conformers in the $LiBF_4$ mixture. The 13 lowest frequency GNMs are shown using the same color coding as in Figure 6b of the main text.

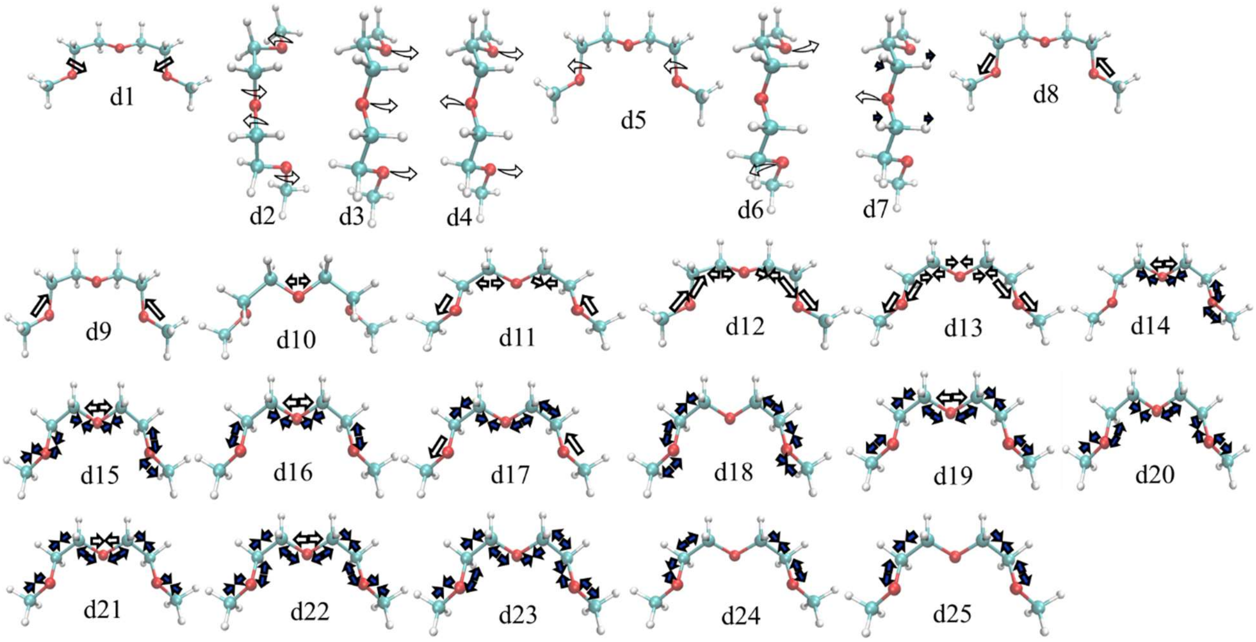

Figure S4. Schematic representations of the generalized normal modes of conformer I ($tg^{+}ttg^{-}t$) of bound diglyme. Curved arrows denote torsional motions, open straight arrows denote bending motions, and filled arrows denote motions of diglyme backbone atoms.

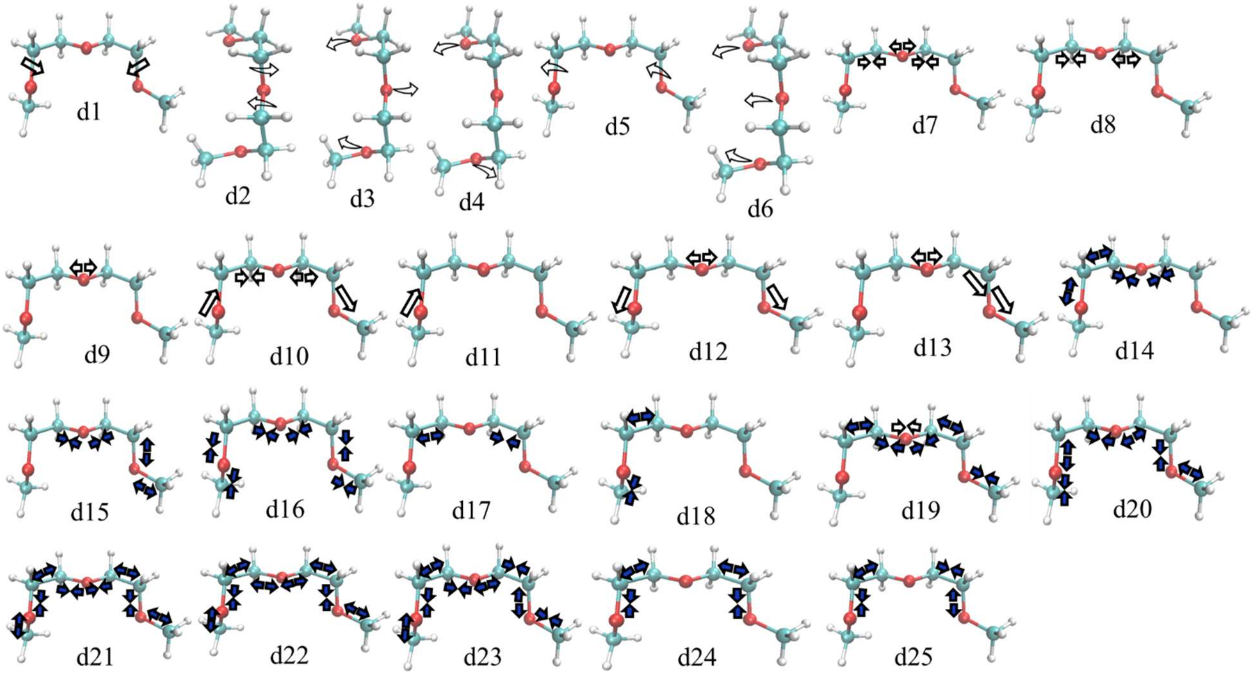

Figure S5. Schematic representations of the generalized normal modes of conformer II (*$tg^+ttg^-g^-$*) of bound diglyme. Curved arrows denote torsional motions, open straight arrows denote bending motions, and filled arrows denote motions of diglyme backbone atoms.

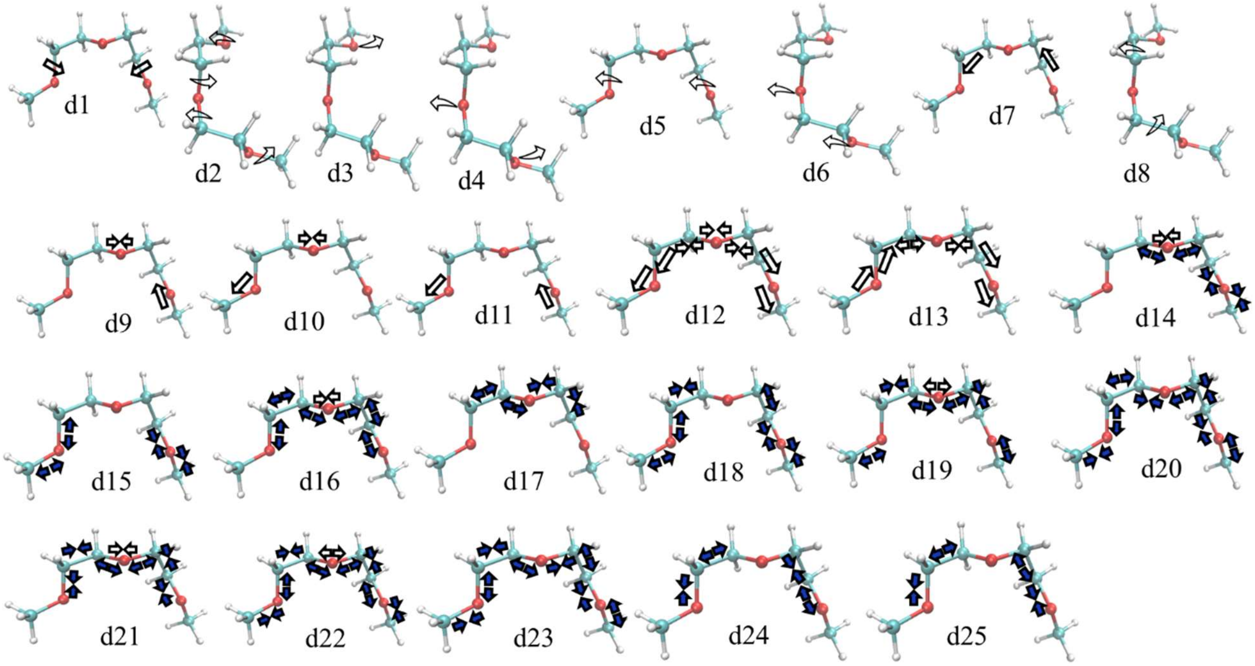

Figure S6. Schematic representations of the generalized normal modes of conformer III (*tg⁻tg⁻g⁻t*) of bound diglyme. Curved arrows denote torsional motions, open straight arrows denote bending motions, and filled arrows denote motions of diglyme backbone atoms.

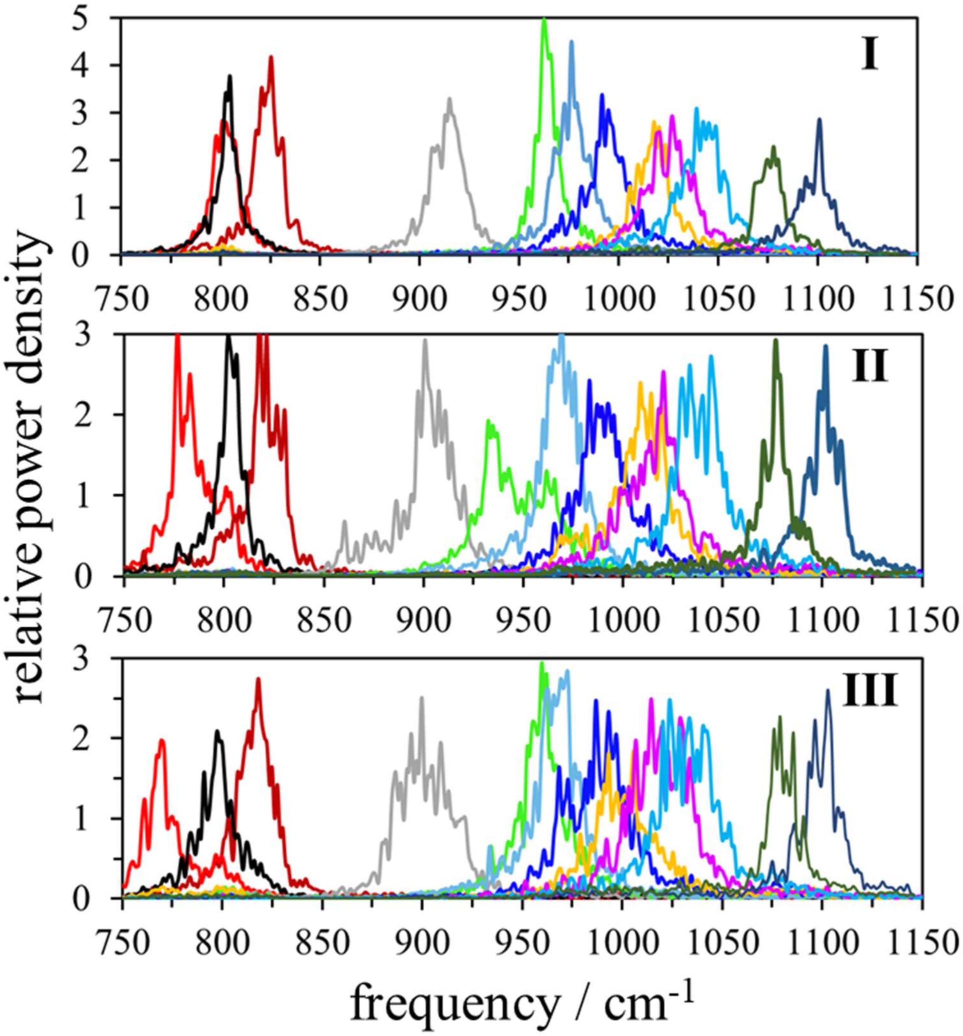

Figure S7. Power spectra of the mid-IR generalized modes of the three most populous bound diglyme conformers in the $LiBF_4$ mixture. GNMs d14-25 are shown using the same color coding as in Figure 8b of the main text.

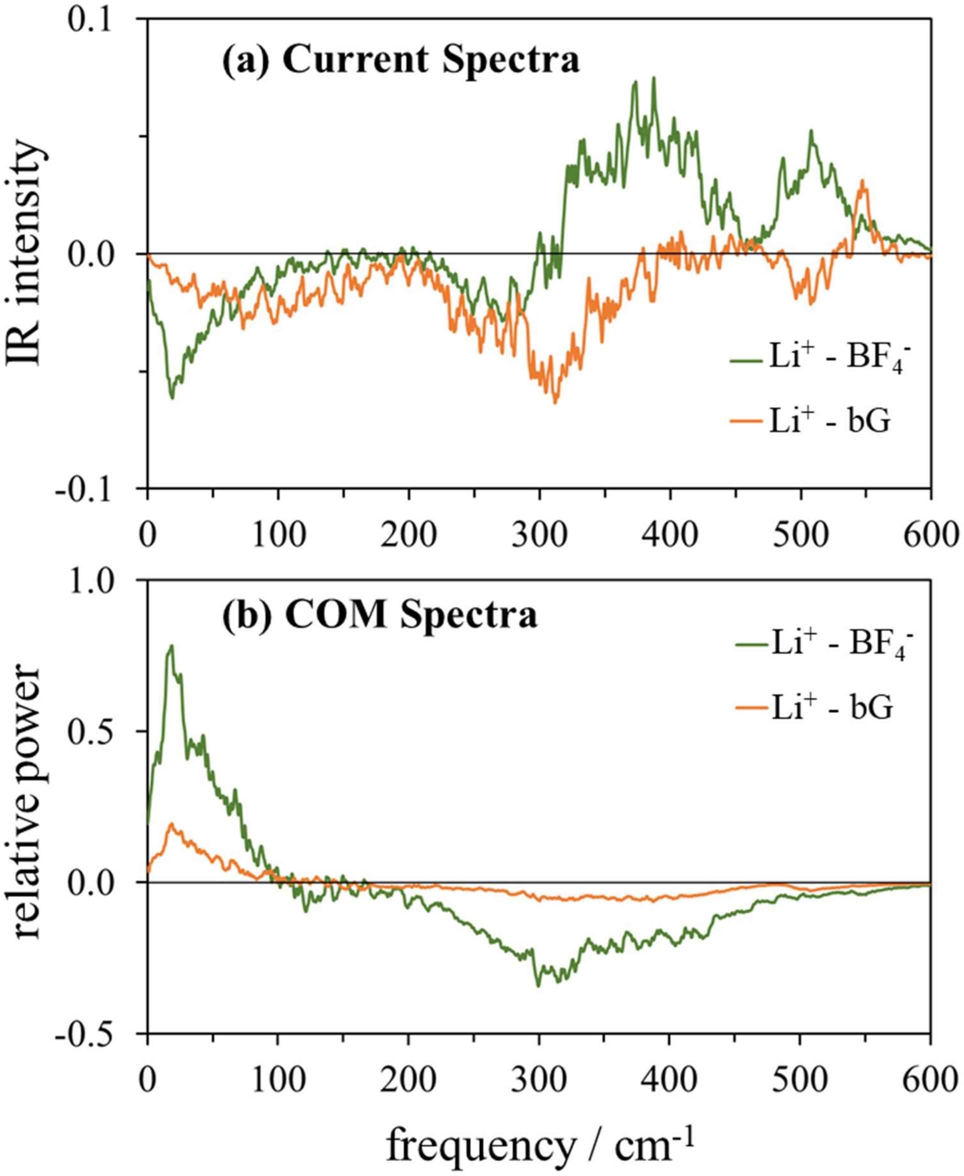

Figure S8. (a) Contributions of current cross-correlations, $\langle \vec{J}_A(0) \cdot \vec{J}_B(t) \rangle$, between Li^+, BF_4^-, and bound diglyme to the IR spectra. (b) Power spectra of the cross-correlation functions of center-of-mass velocities, $\langle \vec{v}_A(0) \cdot \vec{v}_B(t) \rangle$, of these same species.
